# Graphene aerogels: part 2 - derived from commercial graphene and chemically reduced graphene oxide via supercritical carbon dioxide drying

**DOI:** 10.55730/1300-0527.3660

**Published:** 2024-02-08

**Authors:** Meryem SAMANCI, Ayşe BAYRAKÇEKEN

**Affiliations:** 1 Department of Chemical Engineering, Faculty of Engineering, Atatürk University, Erzurum, Turkiye; 2 Department of Nanoscience and Nanoengineering, Atatürk University, Erzurum, Turkiye

**Keywords:** Chemically reduced graphene oxide, carbon aerogel, graphene aerogel, sol-gel method, carbon corrosion, pseudocapacitive charge

## Abstract

Graphene aerogels (GAs), the most important class of carbonaceous aerogels, have attracted attention of many researchers due to their superior physical and chemical properties. In this study, commercial graphene (GR) and chemically reduced graphene oxide (RGO) were used as graphene-based precursor materials, unlike graphene oxide (GO), which is widely used in the literature in GA synthesis. GAs were synthesized using the sol-gel technique and dried with supercritical carbon dioxide (SCCO_2_). In addition, graphene-based materials were used in different ratios and their distribution in the aerogel matrix and its effect on surface properties were investigated. In addition, the synthesized GAs were structurally compared with GR, RGO, and carbon aerogel (CA) without graphene-based materials. Physical characterizations (Brunauer, Emmett, and Teller (BET) analysis, scanning electron microscope-energy dispersive X-ray (SEM-EDX) analysis, micro-Raman spectroscopy, X-ray diffractometer (XRD) were made to examine the structural properties of GAs. In order to analyze the behavior of the surfaces of the synthesized materials against electrochemical corrosion, cyclic voltammetry (CV) and electrochemical impedance spectroscopy (EIS) analyses were performed. As a result of the electrochemical corrosion process of the synthesized materials, the change in their specific capacitance and the formation of pseudocapacitive charge on the surfaces were examined.

## 1. Introduction

Carbonaceous aerogels (carbon [1], carbon nanotube (CNT)-based [2], graphene [3], activated carbon [4], and biomass-derived [5] aerogels) constitute a large class of three-dimensional (3D) carbonaceous materials. Carbonaceous aerogels have superior properties such as high porosity (99%–90%), high surface area, and low density (0.003 gcm^−3^) [6]. They are designable materials because they have controlled structural, textural, and chemical properties. Therefore, they are widely used in many promising areas, primarily as electrodes [1] and catalyst support [3] materials. On the other hand, carbonaceous aerogels form solid substrates with metal oxides [7] or conductive polymers [8], depending on their intended use.

Graphene (GR) is a two-dimensional carbonaceous material with unique electronic, thermal, mechanical, and chemical properties. These features have enabled GR to be used in many application areas. On the other hand, GR has a hydrophobic structure. This property causes poor dispersion in solution mediums or polymeric matrices. However, it exhibits a high tendency to agglomerate due to π-π physical interactions between GR plates [9]. Moreover, large quantities of graphene are very difficult to produce. To overcome these challenges, modified graphenes (graphene oxide (GO), reduced graphene oxide (RGO)) are synthesized [10]. GO, used as a precursor material in the synthesis of graphene aerogel (GA), is generally synthesized by the Hummer method [11]. Unlike GR, GO has functional groups containing oxygen (hydroxyl, aldehyde, and carboxyl groups) in its basal plane. This makes it easier for GO to form composites with different chemical structures. RGO has a structure between GO and GR and is more hydrophobic than GO, with a higher C/O ratio than GO. This characteristic brings RGO closer to the sp^2^ nature of GR sheets. Various methods are used in RGO synthesis [12], including thermal [13,14], chemical [15,16], solvothermal [17,18], electrochemical [19,20], microwave [21,22], and multistep [23,24] reductions. The RGO used in this study was synthesized via chemical reduction. Many reducing agents (hydrazine [25,26], N, N-dimethylformamide (DMF) [26,27], thiourea dioxide [28,29], L-ascorbic acid (LAA) [30,31], hydroquinone [32], sodium borohydride [32,33]) have been used in the chemical reduction process. In this study, hydrazine monohydrate was used [10].

2D graphene-based materials, which have superior physical and chemical properties, offer low porosity despite their high surface area. In order to overcome this situation, a lot of work has been done on GO-derived 3D GA synthesis in general. 3D GA structures are synthesized by covalent bonds formed between the oxygen-containing functional groups on the surface of GO plates and any binding material [34]. Like other carbonaceous aerogels, GAs can be synthesized with different morphological and electrochemically adjusTable properties specific to the areas of use, with changes to be made in synthesis steps and parameters. Jung et al. used the electrochemical exfoliation technique of precursor GOs with different properties (aspect ratio of graphene sheets and porosity of graphene network) that they used in GA synthesis in different electrolyte environments (0.2 M sulfuric acid (H_2_SO_4_) with different concentrations (23, 30, and 37 wt%) of potassium hydroxide (KOH)). GAs with different properties were synthesized by drying the graphene gels they synthesized with GOs of different structures at different freeze-drying temperatures (−200 °C, −80 °C, and −20 °C). The surface areas of GO-derived GAs synthesized with 30% KOH electrolyte at freeze-drying temperatures of −200 °C, −80 °C, and −20 °C were calculated as 315 m^2^g^−1^, 441 m^2^g^−1^, and 504 m^2^g^−1^, respectively. They reached 325 Fg^−1^ capacitance and 45 Whkg^−1^ energy density values with GA obtained at −20 °C freeze-drying temperature [35]. Cheng et al. synthesized GAs using the hydrothermal technique in the presence of sodium hydrogen sulfite (NaHSO_3_) reducing agent with different GO contents. The gels were dried with supercritical ethanol and annealed in N_2_ ambient at 1500 °C. As the GO ratio increased, the thermal conductivity ratios remained in the range of 0.0363–0.0667 Wm^−1^K^−1^ depending on the increase in the bulk density. The increase in thermal conductivity with the increase of bulk density is related to the denser network structure of GAs. In addition, an increase in electrical conductivity was observed as a result of the annealing process. As the GO ratio increased, the electrical conductivity increased within the range of 53.5–157.3 Sm^−1^ [36].

Carbonaceous materials have been studied by researchers in many fields due to their abundance and availability. These materials have many advantages as well as some disadvantages. The most important disadvantage is that the carbon surface is easily exposed to electrochemical corrosion. Much effort has been made and many methods have been applied to mitigate carbon corrosion and increase the material’s resistance to corrosion. At the forefront of these methods is the graphitization of carbon, which enhances electronic conductivity and corrosion resistance [37]. Qiao et al. synthesized a 3D porous graphitic carbon (PGC) derived from polymer hydrogel at lower temperatures (1100 °C) in the presence of an Mn catalyst, in contrast to the graphitization process at very high temperatures (3000 °C). They also reported that they developed a highly durable and active Pt catalyst on this support [37]. Another method against carbon corrosion involves the application of conductive polymer coatings. Jafari et al. studied polyaniline (PANI)/graphene (G) nanocomposite coatings to protect Cu metal from corrosion. PANI/G nanocomposite coating was successfully applied to the Cu surface via electro-deposition technique utilizing cyclic voltammetry in a sulfuric acid environment. PANI/G nanocomposite significantly increased the corrosion protection of Cu metal even in the presence of 5000 ppm NaCl solution [38].

One of the areas affected by carbon corrosion is fuel cells, where chemical energy is converted into electrical energy. Carbonaceous materials are widely used as catalyst support materials, especially in proton exchange membrane fuel cells (PEMFCs). The harsh conditions of PEMFCs accelerate electrochemical carbon oxidation. On the corroded support material surface, Pt nanoparticles tend to aggregate and the electrochemically active surface area (ESCA) of Pt decreases. This situation reduces the performance of the PEMFC. In their study, Oh et al. exposed carbon nanofiber (CNF) to chemical oxidation at different times before using it as a support material in PEMFCs, and they obtained structures with functional groups containing oxygen at different rates. As the chemical oxidation increased, the CNF surface became more hydrophilic. This contributed to the loading of Pt catalysts on the CNF surface. However, the durability of the PEMFC was reduced because the chemical oxidation process accelerated the electrochemical carbon corrosion and reduced the ESCA of Pt [39]. Berthon-Fabry et al. investigated the effect of fluorination on the durability of CA-supported Pt catalysts to be used as cathodes in PEMFCs. Xenon difluoride (XeF_2_) was used as a fluorine precursor. Fluoridation changed the morphology by increasing the pore size of CA and decreasing the specific surface area. With fluoridation, C-F bonds are formed both with the carbon itself and with the oxygen-containing regions formed during corrosion. The fluoridation step was performed before and after Pt loading on the support material. Fluoridation after Pt loading caused amorphous platinum, which was quite ineffective against the oxygen reduction reaction while facilitating the fluoridation mechanism. It has been reported that the electrochemical resistance is higher in fluorination before the Pt loading process [40].

In numerous studies in the literature, GO was used as a graphene-based precursor material in the synthesis of GAs. In this study, different from the literature, commercial GR and chemical-RGO were used at different rates as graphene-based precursor materials in the synthesis of GAs. Synthesis of GAs was carried out by sol-gel technique, SCCO_2_ drying, and then pyrolysis process. In this way, changes in the physical and electrochemical properties of GAs were investigated by using graphene-based precursor materials (GR and chemically RGO). The synthesized GAs were compared with CA synthesized with graphene-based precursor materials and the reactant concentration was used at the same rate without graphene. The synthesized materials underwent physical analyses, including Brunauer, Emmett, and Teller (BET) analysis, scanning electron microscope-energy dispersive X-Ray (SEM-EDX) analysis, micro-Raman spectroscopy, X-ray diffractometer (XRD). Cyclic voltammetry (CV) analyses and electrochemical impedance spectroscopy (EIS) was performed to examine the electrochemical behavior of the synthesized GAs against carbon corrosion.

## 2. Materials and methods

### 2.1. Experimental materials

Graphite (flake, 325 mesh, Alfa Aesar), phosphorus pentoxide (P_2_O_5_, ≥ 98%, Sigma-Aldrich), potassium persulfate (K_2_S_2_O_8_, ≥ 99.9%, Sigma-Aldrich), potassium permanganate (KMnO_4_, 99%, Merck), sodium nitrate (NaNO_3_, ≥ 99.9%, Sigma-Aldrich), sulfuric acid (H_2_SO_4_, 95%–98%, Merck), hydrogen peroxide (H_2_O_2_, 30%, Merck), and hydrochloric acid (HCl, 37%, ISOLAB) were used for GO synthesis. During the RGO synthesis process, hydrazine monohydrate (NH_2_NH_2_·H_2_O, Sigma-Aldrich) and methanol (CH_3_OH, VWR CHEMICALS) were used. Commercial graphene (GR, 5XG Sciences, xGnP® Grade C) was used for the synthesis of GAs.

Pure water, 99% resorcinol (C_6_H_4_(OH)_2_, Sigma-Aldrich), 34.5% formaldehyde (CH_2_O, Sigma-Aldrich), and as catalysts, 99% sodium carbonate (Na_2_CO_3_, Sigma-Aldrich) and acetone (99.5%, Sigma Aldrich) were used for carbon and GA synthesis. Carbon dioxide (CO_2_, Habaş Company) was used as the supercritical fluid.

The electrode ink consisted of Nafion solution (15%, Ion Power, Inc.), 1,2-propanediol (C_3_H_8_O_2_, ≥ 99.5%, Sigma-Aldrich), and the corresponding material. Perchloric acid (HClO_4_, 70%–72%, ISOLAB) was used as the electrolyte. High-purity nitrogen (N_2_, Habaş Company) was used to provide an inert environment.

### 2.2. Synthesis of GO and RGO

GO and RGO syntheses are detailed in our previous studies [10]. GO synthesis was carried out using the Hummer method [41], with commercial graphite utilized as the precursor material. Before proceeding to the GO synthesis with the Hummer method, the graphite was preoxidized. In this way, the exfoliation of graphene is facilitated and the efficiency of the reaction is increased.

RGO synthesis was carried out using GO precursor material and achieved through chemical reduction [10]. The reduction process took place in a reflux system, employing hydrazine monohydrate as the reducing agent. A solution was prepared with predetermined amounts of GO and distilled water. Hydrazine monohydrate was added to this solution and mixed for 6 h at 100 °C and 18 h at its own temperature for a total of 24 h. After filtration, washing was conducted in a centrifuge with methanol and then with distilled water. It was kept in an oven at 80 °C overnight. After synthesis, the brown color of GO changed to black. This is due to the fact that the oxygen-containing functional groups on the surface of the GO plates are considerably reduced by the reduction process.

### 2.3. Carbon aerogel and graphene aerogel synthesis

Aerogels are prepared by polymerization of resorcinol (R) and formaldehyde (F) in the presence of a catalyst (C) using the sol-gel method [1]. Sodium carbonate was used as the catalyst. The sol solution for carbon aerogel synthesis was prepared using 5 mL of distilled water (W) with molar ratios of R/C:100, R/W: 0.02, and R/F:0.5. This sol solution was placed in a glass tube and left at room temperature for 24 h. Subsequently, it was kept in an oven at 50 °C for 24 h, followed by 90 °C for 72 h.

For the synthesis of GAs, GR or RGO (at a ratio of material to total mass of 0.25% (GA-1 or RGOA-1), 0.5% (GA-2 or RGOA-2), and 1% (GA-3 or RGOA-3)) was mixed with 20 mL of distilled water in a beaker for 30 min. Next, reactants (R, F, C) were added to this mixture at the rates specified in carbon aerogel synthesis (the molar ratios of R/C: 100, R/W: 0.02, and R/F: 0.5). The sol solution was stirred for 6 h at approximately 35 °C, with no contact with the environment. The mixture was placed in a sealed tube and kept in an oven for 24 h at 50 °C and then 72 h at 90 °C.

The carbon and graphene gels removed from the oven were taken out of the tube and immersed in acetone, and solvent exchange was performed. They were left in acetone for 48 h. The purpose of doing this is to facilitate supercritical drying. Excess water and catalyst were removed from the pores and replaced with acetone with low surface tension. A high-pressure reactor with a volume of 54 mL was used for the supercritical drying process. During drying, the reactor was initially filled with acetone to prevent porous precipitation and then the wet gel was placed in the reactor. With the help of the syringe pump connected to the system, carbon dioxide was sent to the system at the desired pressure. Samples placed in the reactor to obtain R-F aerogel were extracted with SCCO_2_ at a temperature of 50 °C and a pressure of 17.24 MPa. Finally, R-F aerogel was pyrolyzed in a tubular furnace to obtain CA and GAs (GRAs or RGOAs) structures. N_2_ gas was used to provide an inert atmosphere environment. Before the temperature was increased, N_2_ gas was introduced into the tubular tube for 30 min. CA and GAs (GRAs or RGOAs) synthesis was carried out by pyrolyzing R-F aerogels at 1000 °C, under a N_2_ environment, with a heating rate of 15 °C min^−1^ for 4 h. During the pyrolysis process, excess reactants and residues in the organic aerogel structure are removed, turning the carbon aerogel into a relatively pure carbon structure. With this process, the activation of the synthesized materials and the increase of the surface area are provided.

## 3. Characterization

### 3.1. Physical characterizations of materials

Various characterization methods were used for the characterization of the synthesized materials. Brunauer, Emmett, and Teller (BET, Micromeritics 3Flex 3-port BET surface area device) method and N_2_ adsorption/desorption isotherm analysis were used to determine the surface area, porosity, and pore size distributions of the materials. Morphological structures of the materials were determined by using scanning electron microscope-energy dispersive X-ray analysis (SEM-EDX, Zeiss Sigma 300, magnification ratio: 10×−1.000.000×). Raman spectroscopy (WITech alpha 300R, type of display: 2D-3D, wavelength range: 350–1050 nm) was used to obtain information about the bonds formed for determining order/disorder or stacking of the materials. Crystal structures of the materials were determined by using X-ray diffractometry (XRD, PANalytical Empyrean) in the range of 10° ≤ 2θ ≤ 90°.

### 3.2. Electrochemical characterization of the materials

Electrochemical characterization of RGO, GR, CA, and GAs was performed using cyclic voltammetry (CV), employing the Versastat 3 potentiostat-rotating disc electrode system from Pine Instrument. A standard three-electrode system (comprising working (glassy carbon; GC, 0.1963 cm^2^), reference (Ag/AgCl), and counter (Pt wire) electrodes) was used for CV analysis. The electrolyte used was 0.1 M perchloric acid (HClO_4_).The electrode ink (5 μL for the working electrode) was composed of the material synthesized at a mass loading rate of 28 μgcm^−2^, 1 mL of distilled water, 1 mL of 1,2-propanediol, and 165 μL of 15% Nafion solution. N_2_ gas and the test medium were ensured to be inert during the measurements and during the corrosion process. The working electrode was subjected to 24 h corrosion treatment (Applied DC: 1.2 V). Electrochemical impedance spectroscopy (EIS) analyzes was carried out. Nyquist plots (imaginary impedance - real impedance) were used to determine the behaviour of the materials before and after the corrosion.

## 4. Results and discussion

Physical characterizations for graphite, synthesized GO and RGO are given in our previous study [10]. BET, SEM-EDX, XRD, and RAMAN analyses were conducted for GRAs and RGOAs.

**Figure 1:**
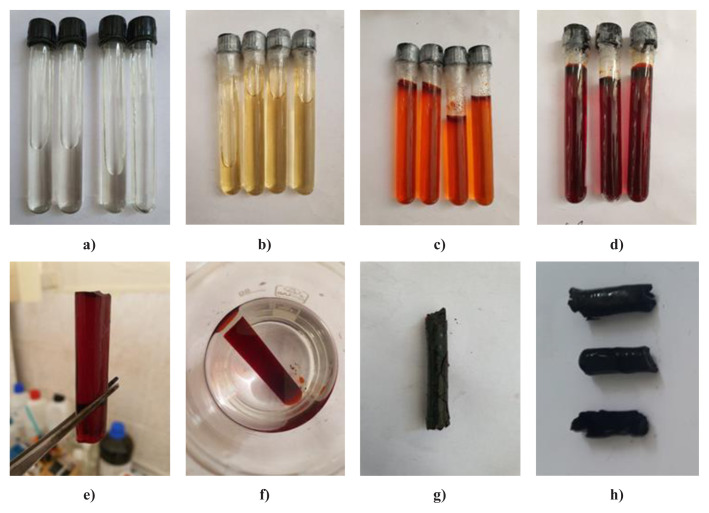
Digital photographs of the synthesis stages of CA, **a)** sol solution of CA, **b)** Gel kept at room temperature for 24 h, **c)** gel kept at 50 °C for 24 h, **d)** gel kept at 90 °C for 72 h, **e)** gel removed from the glass tube, **f)** gel thrown into acetone (48 h), **g)** After supercritical drying (R-F aerogel), **h)** after pyrolysis (CA).

**Figure 2:**
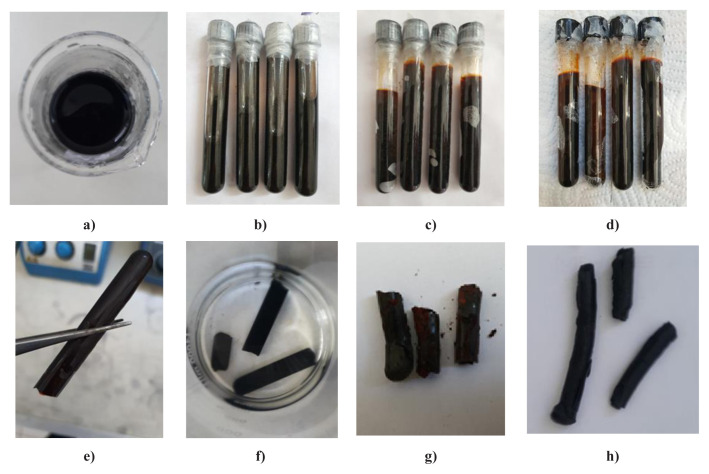
Digital photographs of the synthesis stages of GAs, **a)** sol solution of GAs, **b)** sol solution mixed at 35 °C for 6 h, **c)** gel kept at 50 °C for 24 h, **d)** gel kept at 90 °C for 72 h, **e)** gel removed from the glass tube, **f)** gel thrown into acetone (48 h), **g)** after supercritical drying (R-F aerogel), **h)** after pyrolysis (GRAs or RGOAs).

Digital photographs of the synthesis stages of CA, GRAs, and RGOA synthesized with the sol-gel method are shown in Figures 1 and 2. The sol solution of CA transitioned from transparent color to brown upon gelation. Following SCCO_2_ drying and subsequent pyrolysis, the gel turned black, typical color of carbonaceous materials. Similarly, in the synthesis stages of GRAs and RGOAs, the colors observed—black in the sol solution, during gelation, SCCO_2_ drying, and pyrolysis stages—are attributed to the presence of graphene-based precursor materials (GR, RGO) in the sol solution.

The BET method and N_2_ adsorption/desorption isotherm analysis were used to determine the structural properties of the materials, as presented in Table 1. The surface area of the GR was measured as 710.34 m^2^g^−1^. In our previous study, GO was synthesized via the Hummer method using commercial graphite powder [10]. As a result of the synthesis, the graphite layers were effectively separated into thin flakes, and functional groups containing oxygen were located on the graphene basal plane and corners. The surface area of GO was measured as 10.51 m^2^g^−1^. RGO structure was obtained by the GO chemical reduction process. In this way, the amount of oxygen-containing functional groups in the structure of GO has been reduced. The surface area of RGO was measured as 166.92 m^2^g^−1^. RGO had a structure between GR and GO [10]. Graphene-based materials with varying surface properties have different degrees of hydrophilicity or hydrophobicity. Therefore, differences occurred in the dispersion properties of GR and RGO in the sol solution during sol-gel synthesis and in cross-linking mechanisms in the gel [42].

When GR was added to the sol solution while synthesizing GAs, the surface area first decreased compared to CA (202.86 m^2^g^−1^). As observed in the EDX analysis, the amount of oxygen-containing functional groups on the surface of the GR plates is considerably lower than that of RGO. Therefore, commercial GR exhibits greater hydrophobicity than RGO [43]. The mean pore diameter of GRAs is 4 nm, while the mean pore diameter of RGOA is 3 nm. This shows that, due to the structural properties of the graphene precursor material, the covalent bonding mechanism is dominant in RGOAs, while π-π interactions are dominant in GRAs. The average particle size distributions of GAs also differ. In GRAs, GR sheets tend to agglomerate in the aerogel with the lowest GR ratio, while in the aerogel with the highest GR ratio, the GR sheets are dispersed within the structure to form a network. In other words, as the GR ratio in the aerogel increased, the average particle size decreased and accordingly the surface area increased (GA-3, 471.88 m^2^g^−1^). In addition, the high surface area of commercial GR also contributed to increasing the surface area of GRA as the GR ratio increased within the structure. When RGO was added to the sol solution, the surface area first increased considerably compared to CA. This is attributed to the higher hydrophilicity of RGO compared to GR, due to the presence of oxygen-containing functional groups in its structure. These functional groups on the surface of the RGO plates facilitate the coating of the R-F hydroxymethyl derivatives on the surface of the graphene planes as polymer chains [34]. This improved the surface area of GAs. As the ratio of RGO in the aerogel increased, the particle size increased. Due to the increase in volume of the RGO sheets due to the effect of the functional groups on the RGO surface, there was a corresponding increase in agglomeration tendency as the RGO ratio in the aerogel increased, resulting in a decrease in surface area. As the amount of RGO increased, the surface area decreased from 511.75 m^2^g^−1^ (RGOA-1) to 224.75 m^2^g^−1^ (RGOA-3) due to insufficient R-F polymer chains. These alterations in the surface area properties of GRAs and RGOAs are also clearly demonstrated by SEM images.

**Figure 3:**
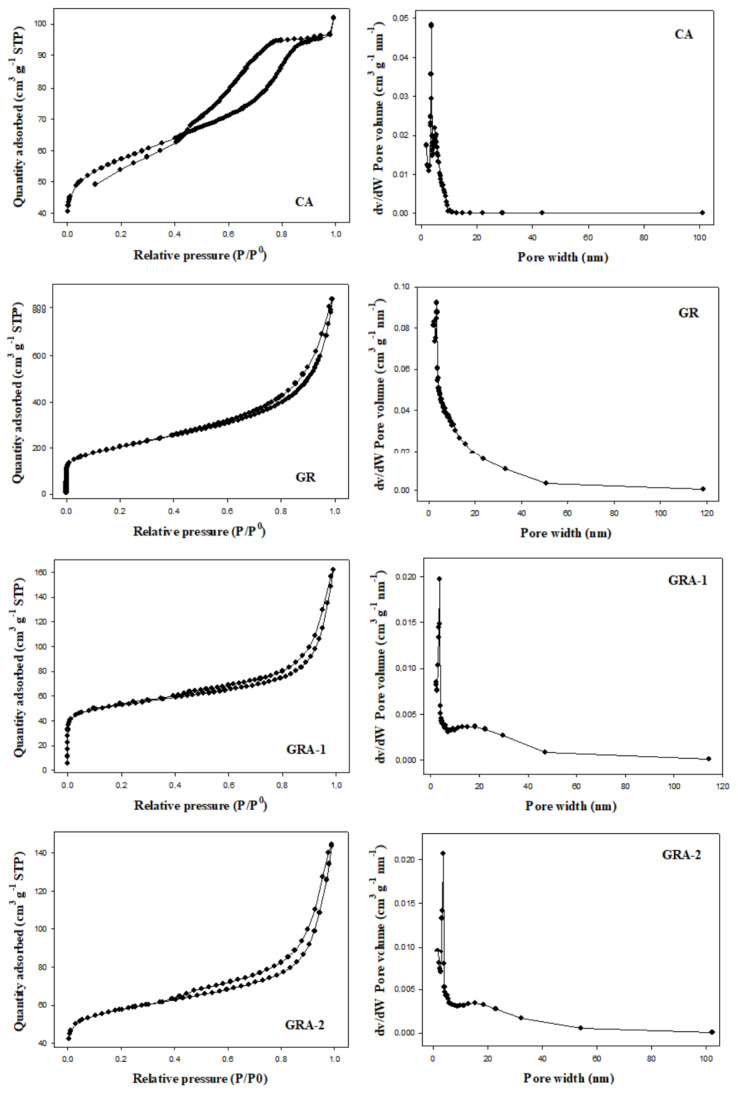

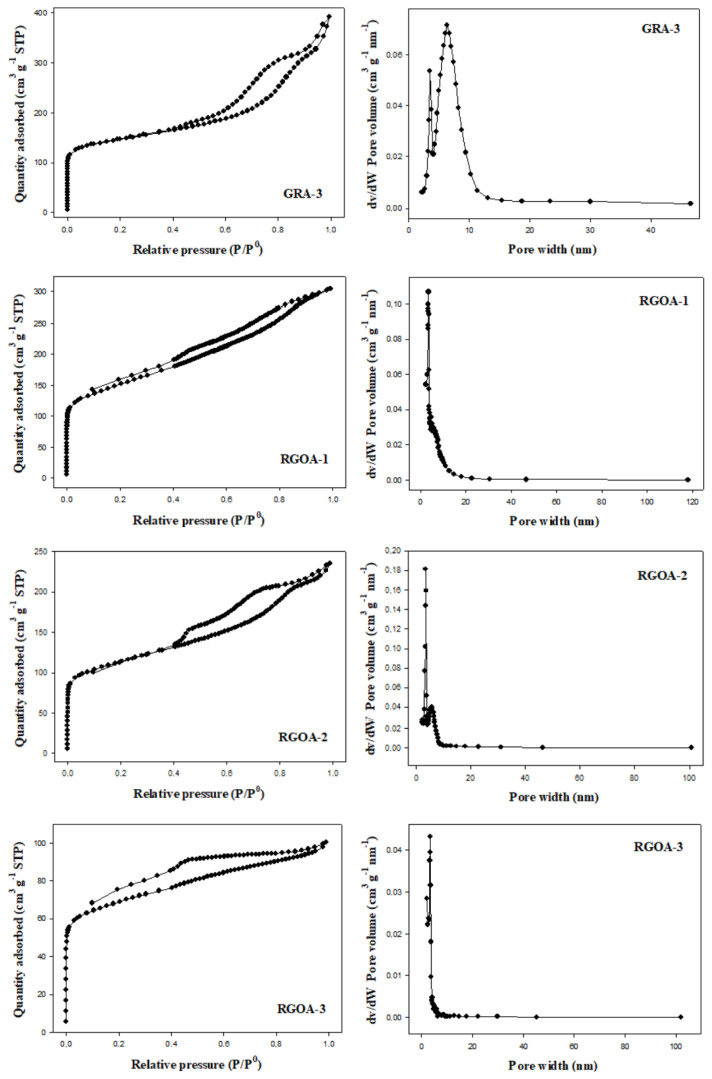
N_2_ adsorption-desorption isotherms and pore size distributions of GR, CA, GRAs, and RGOAs.

N_2_ adsorption/desorption isotherms and Barrett-Joyner-Halenda (BJH) pore size distributions of the synthesized materials were analyzed and the results are presented in Figure 3. Adsorption isotherms observed experimentally by IUPAC are classified into 6 different types, while hysteresis loops are classified into 4 different types [44–46]. The CA structure corresponds to the type IV isotherm and the H2 hysteresis. This situation is generally explained as mesoporous and materials with irregular pore size and shape distribution [47]. GR and GAs correspond to type IV isotherm and H3 hysteresis. They are described as structures in which plate-like particles lead to slit-shaped mesopores [48]. RGOAs correspond to type IV isotherm and H4 hysteresis. This is explained by the presence of micropores in the mesoporous structure [49]. As a result, changes in graphene-based precursor materials caused differences in pore size distributions and pore shapes of graphene aerogels.

**Figure 4:**
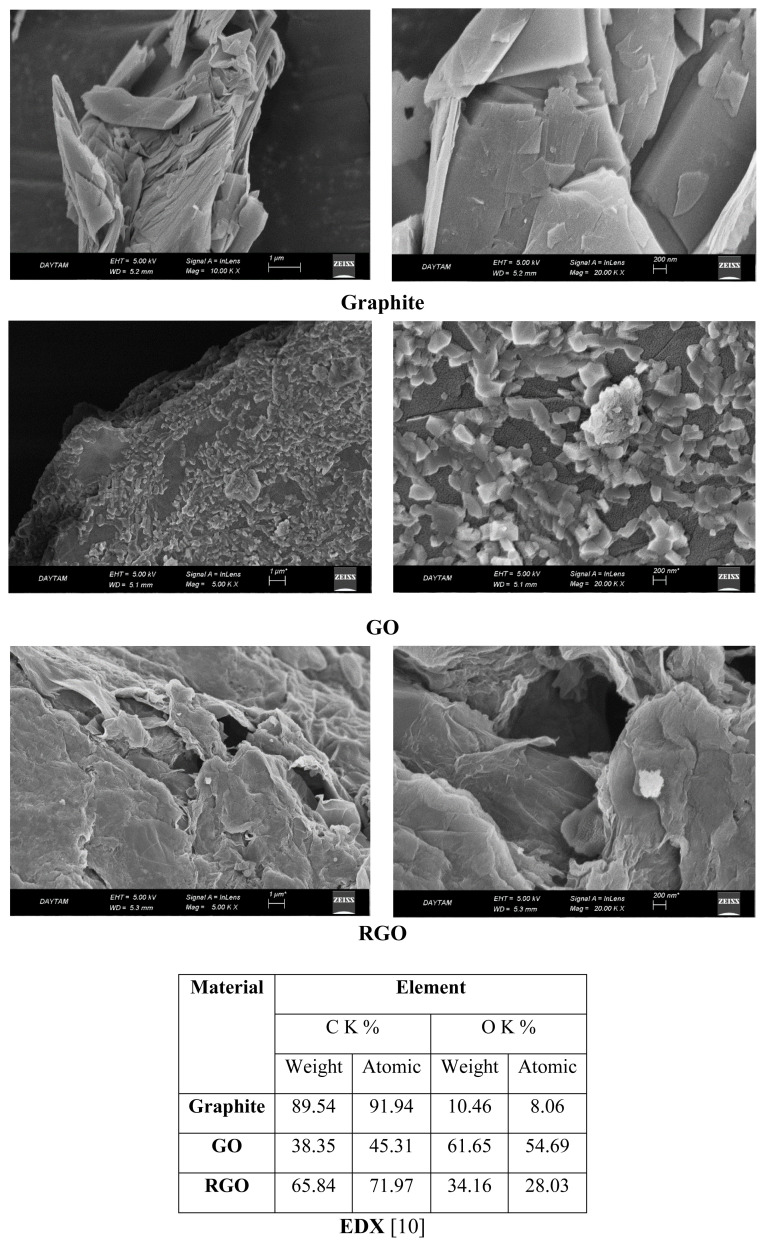
SEM images and EDX analyses of commercial graphite, GO, and RGO.

**Figure 5:**
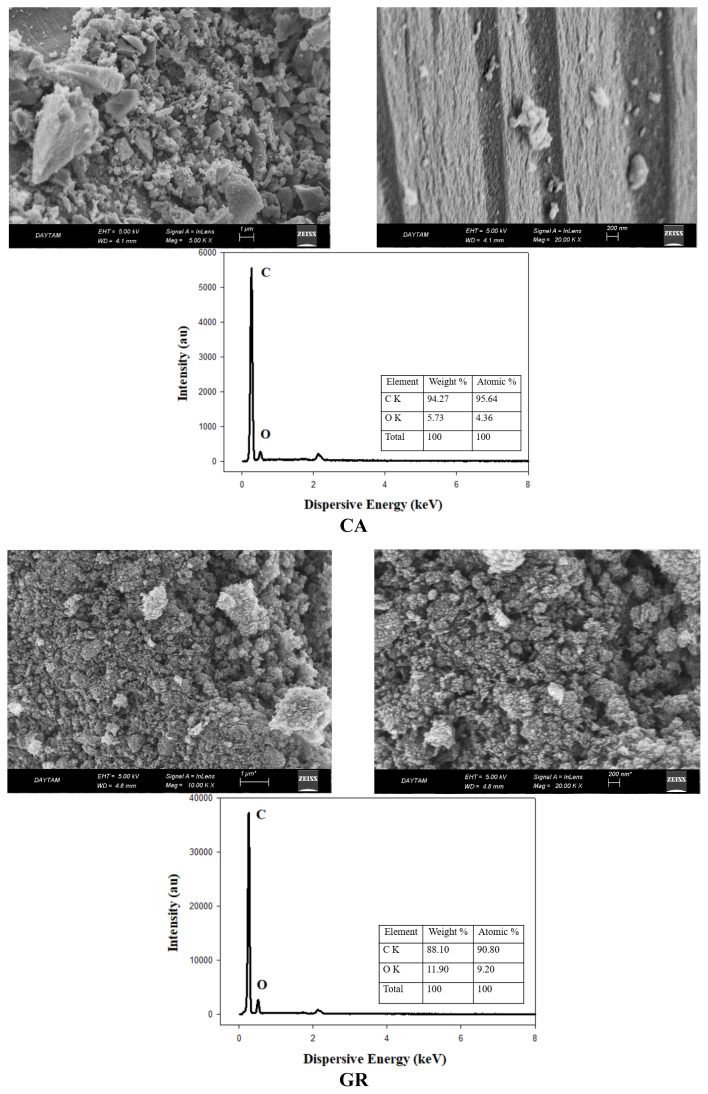

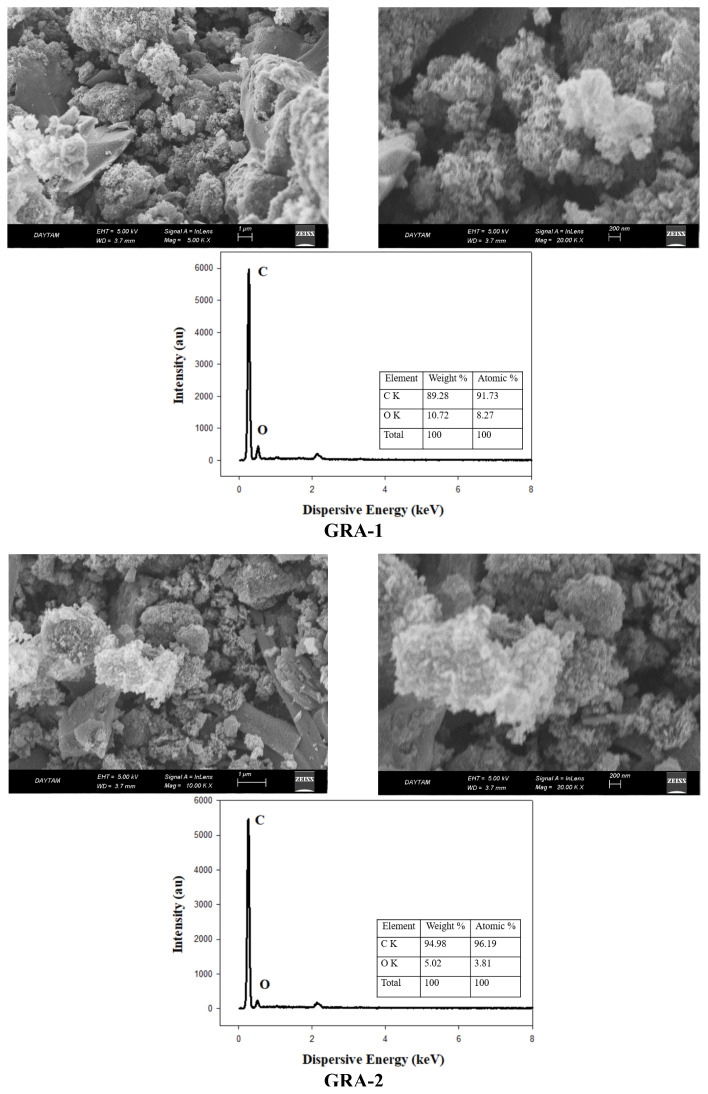

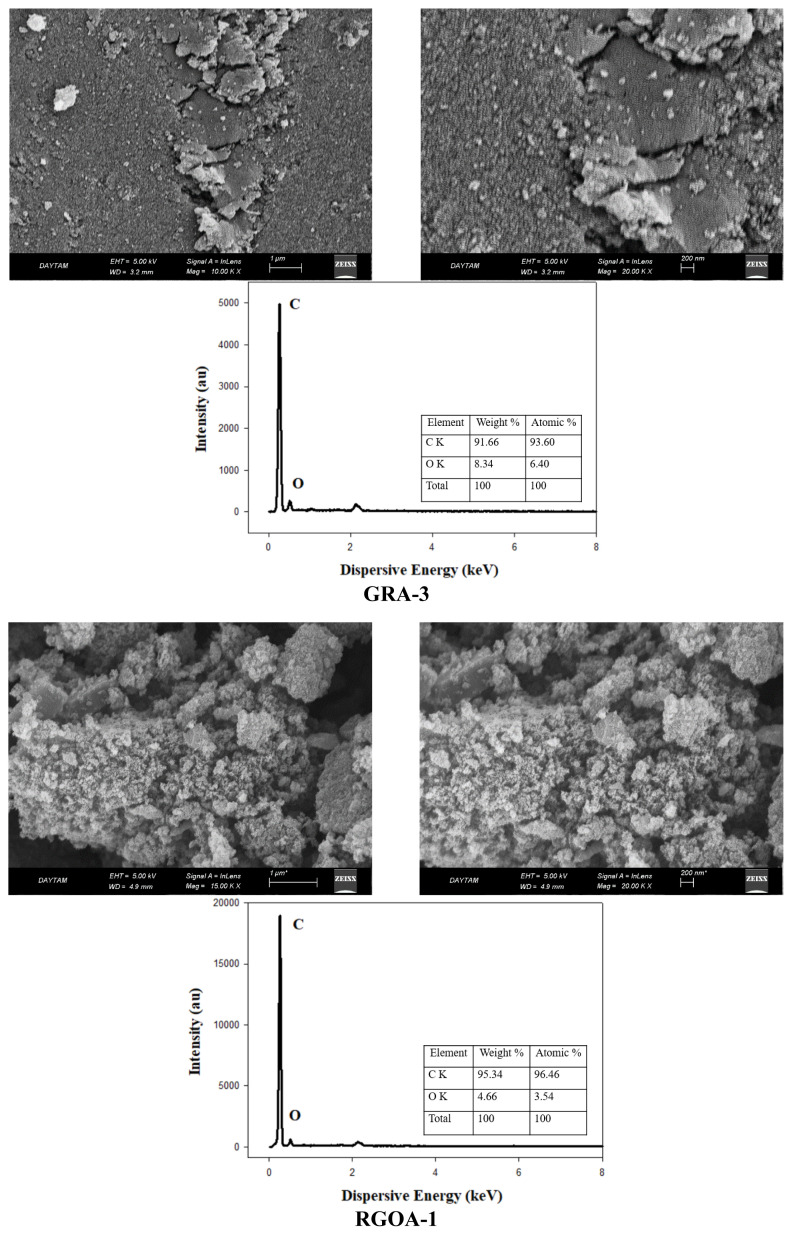

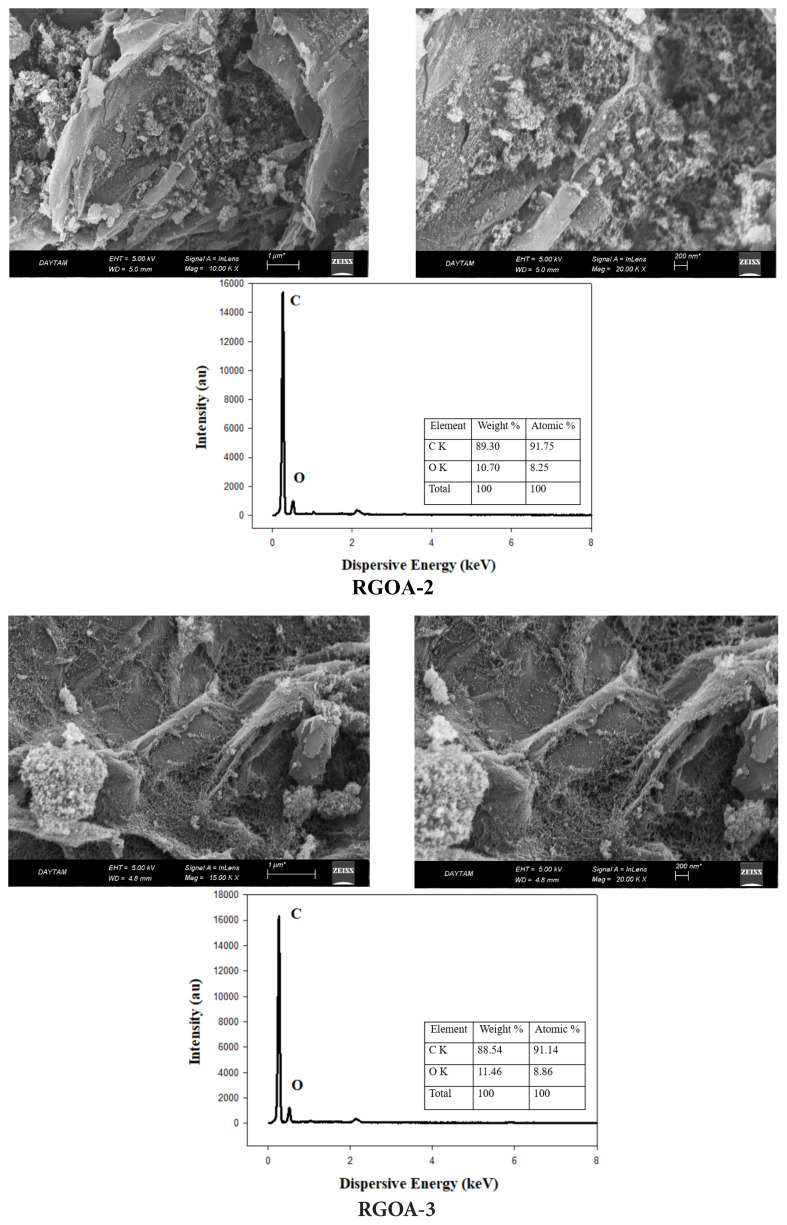
SEM images and EDX analyses of GR, CA, GRAs, and RGOAs.

SEM images of commercial graphite, GO, and RGO are given in Figure 4. In the SEM images, the image of graphite as graphene stacks is clearly seen. GO synthesis was carried out by exfoliating graphite. It is seen that the graphene plates get smaller and the number of layers decreases. The RGO structure was obtained by chemical reduction of GO [10]. The surface of graphene plates is wider than graphite. In the EDX analysis provided in Figure 4, the weight oxygen ratio in graphite, GO, and RGO structures is given as 10.46%, 61.65%, and 34.16%, respectively [10]. This shows that the oxygen-containing functional groups placed between the graphene plates are considerably reduced by the chemical reduction process. It is seen that the synthesized RGO reaches a structure between GR and GO. These changes in graphene-based materials can also be explained by the BET results presented in Table 1. The distribution of modified graphenes of different structures in the sol solution is also different. Consequently, a wide variety of GAs with different surface properties were synthesized, as can be clearly seen from the SEM images given in Figure 5.

During the synthesis of GAs, variations in the cross-linking mechanisms and gelation processes occurred, influenced by the structural properties and the amount of graphene-based precursor materials used in the sol solution. The change in the structure and amounts of GR and RGO significantly altered the final aerogel structures. Observing the SEM images of GRAs and RGOAs, it is seen that the aerogels have a structure between CA and graphene-based precursor material. As the ratio of graphene-based material in the aerogel increases, it is seen that the R-F hydroxymethyl polymer chains are insufficient to coat the graphene plate surfaces. This is due to the fact that graphene-based materials, although very light, occupy too much volume in the R-F sol matrix. In the case of 0.25% doping of graphene-based precursor materials, GR and RGO were embedded in the structure. As the amount of GR and RGO increased, graphene plates in the aerogel structure began to appear [34]. Thus, the 3D network structure of GAs and the formation of micro- and mesopores with randomly cross-linked graphene layers were realized [50]. Lim et al., using acetonitrile as GO precursor and an acid catalyst, synthesized GO-RFA-P aerogel very quickly with the sol-gel method after supercritical drying and pyrolysis steps. The SEM analysis of GO-RFA-P reveals a structure comprising randomly oriented graphene sheets several microns thick, embedded in a network of pyrolyzed R-F condensate chains [51]. dos Santos-Gómez et al. synthesized GAs with different GO contents, which exhibited morphological differences depending on whether they were catalyzed or not during synthesis. They reported that in catalyzed aerogels, the graphene sheets were randomly dispersed in the presence of low GO, and the porosity of the mesh increased due to the rolling-folded graphene sheets at high GO ratios. On the other hand, they showed that uncatalyzed GAs have larger macropores and smoother and thinner wall structures than catalyzed ones [34].

According to the EDX results of GR, CA, GRAs, and RGOAs, the weight C ratios of the materials vary between 88% and 95%. In particular, the weight and atomic carbon ratios of RGOA have increased considerably compared to the RGO precursor material. In this case, it is seen that the oxygen in the aerogel is significantly removed by the carbonization process. Owing to the presence of oxygen-containing functional groups in RGO, the oxygen ratio increased as the RGO ratio in RGOs increased. Nagy et al. synthesized polymer aerogels with different GO ratios. Carbonization of these polymer aerogels was achieved by pyrolyzing them at 800 °C in a N_2_ atmosphere. The surface area of the resulting carbonaceous aerogels increased by 25% due to the considerable reduction in the content of oxygen-containing functional groups in the content of GO in the aerogel structures during the pyrolysis process. As a matter of fact, after carbonization, the C/O atomic ratio of GO increased from 2 to 4 [52]. The C/O ratios obtained as a result of EDX analysis of graphene-based materials and GAs are given in Table 2. The C/O ratio of GAs is considerably increased compared to GR and RGO precursor materials. The aerogel with the highest C/O ratio belongs to RGOA-1 with 20.46 by weight. Oxygen-containing functional groups, separated in the aerogel structure with the pyrolysis process, increased the C/O ratio. Due to the porous structure formed as a result of this, surface areas have also been developed.

**Figure 6:**
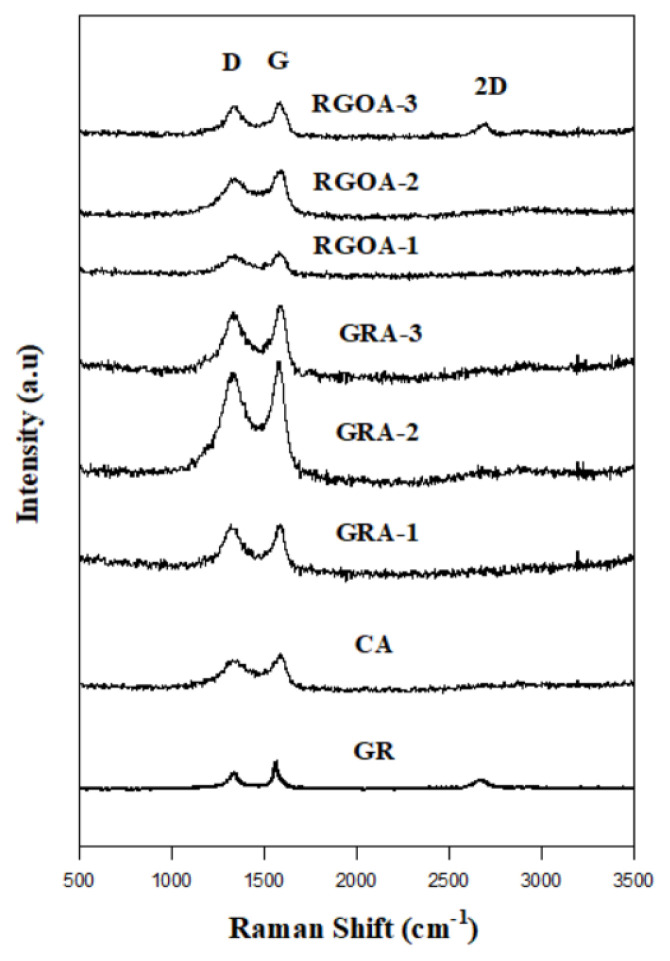
Raman spectra of GR, CA, GRAs, and RGOAs.

Raman spectra of the synthesized materials are given in Figure 6. The analysis results of these spectra are summarized in Table 3. The D-band is related to the defective regions caused by the oxygen-containing functional groups, while the G-band is related to the carbon regions where sp^2^ hybridizations occur. On the other hand, the frequency region where the G-band is located is related to the layers of graphite or graphene sheets. Accordingly, the intensity ratios of the D and G-bands explain the average size of the carbon fields where sp^2^ hybridizations occur [53]. Xu et al. prepared wet hydrogels by in situ chemical reduction using GO-derived, L-phenylalanine as the reducing agent. Subsequently, they obtained GA structures by freeze-drying. The G bands of GO and GA were found to be 1599 cm^−1^ and 1587 cm^−1^, respectively. The frequency difference of 12 cm^−1^ indicates that GO is efficiently reduced by L-phenylalanine. Also, the ID/IG ratios of GO and GA are 1.8 and 1.6, respectively. This indicates that the average size of sp^2^ hybridization domains of GA increased after chemical reduction [54].

It is seen that the frequency values of the D and G bands of GAs are observed to be very close to each other. This is due to the fact that GAs and graphene layer numbers are close to each other. It is also clearly seen in the analysis results of the XRD spectra given in Table 4. On the other hand, the intensity values of the D and G bands differ considerably. Compared with GR, the number of defects in graphene sheets of GRAs increased. When compared with RGO, the number of defects in the graphene sheets of RGOAs decreased [55]. The aerogels with the most intense sp^2^ hybridization areas belong to GRA-2 and RGOA-2 with I_D_/I_G_ ratios of 0.54 and 0.69, respectively.

Generally, graphene-based materials have a 2D peak in 2500 cm^−1^ to 3000 cm^−1^ Raman shift region. The overtone peak of D is a 2D peak, representing the number of layers. This 2D peak is effective not only in the presence of defects but also in their absence, and its density decreases with defects [56]. On the other hand, the number of layers of the obtained material can be determined from the position of the 2D band. In this case, the smaller the 2D vertex position, the lower the number of layers and the wider the vertex [57]. The 2D peaks of the graphene-based precursor materials (GR and RGO [10]) were prominent, while the 2D peaks of the GAs were almost insignificant. This is explained by the structural similarity of GAs to conventional CAs [58]. The 2D peak was evident when the amount of GR or RGO in the GAs was 1%. This 2D peak shows that a small amount of graphene-based plates in the GA are homogeneously dispersed in the aerogel without deteriorating its structure. This is also proven by SEM images.

Additionally, the average in-plane crystal size (L_a_) of the synthesized materials was calculated using the Raman data in Table 3. Equation 1 was used for this calculation [59].


(1) $$L_{a}=\frac{44}{\left(ID/IG\right)}$$

**Figure 7:**
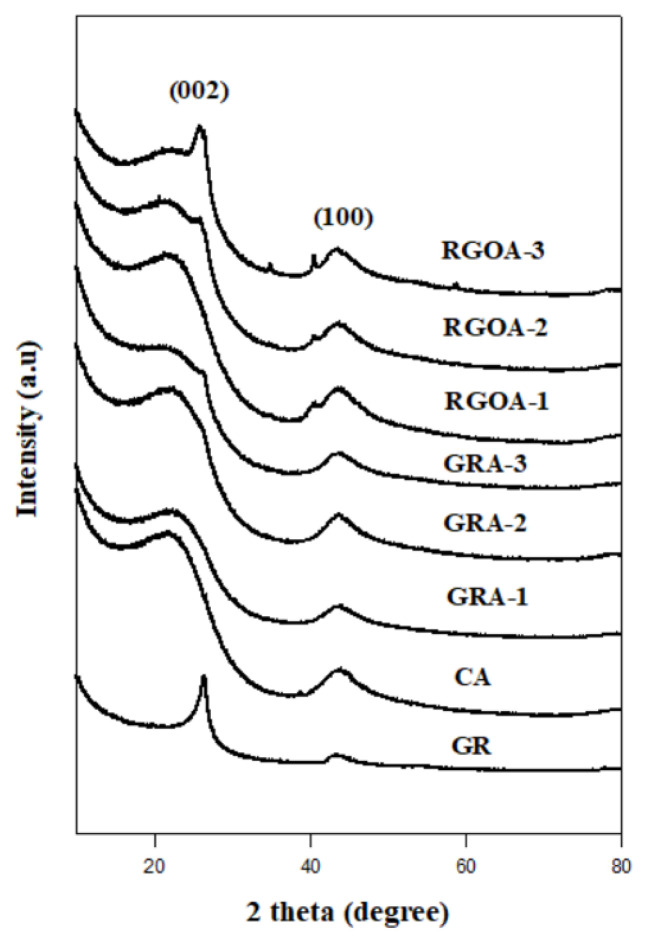
XRD spectra of GR, CA, GRAs, and RGOAs

The XRD spectra of the synthesized materials are given in Figure 7 and the analyses of the data of the spectra are given in Table 4. There are two distinct peaks belonging to the (002) and (100) planes. These planes approximately belong to the angles 2θ = 22° and 2θ = 43°, respectively. The most prominent (002) plane represents graphitic-like structures [60]. This broad (002) peak indicates disordered graphene nanosheets along their stacking direction. This supports that the aerogel network consists of several layers of stacked graphene sheets, which is consistent with the SEM images [61]. On the other hand, RGOAs have a few small peaks between the (002) and (100) planes. These peaks became more pronounced as the amount of RGO in RGOA increased. This is due to the presence of graphene plate surfaces, which are excessive for R-F polymerization during the gelation process, due to the increased amount of RGO in the sol solution. These differences in cross-linking mechanisms during the gelation process are also clearly demonstrated by BET analyses, SEM images, and Raman spectra.

The results of the peak analyses of the XRD spectra given in Figure 7 are summarized in Table 4. The concept of interlayer distance for graphene-based materials is based on Bragg’s law and is calculated from the Sherrer equation [62,63].


(2) $$n\lambda =2d_{\left(hkl\right)}\;\text{sin }\theta$$

(3) $$L_{c}=\frac{0.89\lambda }{\beta_{c}\;\text{cos }\theta }$$

(4) $$L_{a}=\frac{1.84\lambda }{\beta_{a}\;\text{cos }\theta }$$

(5) $$N=\frac{L}{d_{\left(hkl\right)}}$$

Here, λ is the wavelength (1.5016 Å) of the X-ray, θ is the scattering angle, n is an integer representing the order of the diffraction peak, d_(hkl)_ is the distance between the layers, (hkl) is the Miller indices, L_c_ is the stacking height (vertical size of crystallites, for the (002) peak), L_a_ is the lateral size of the crystallite (for the (100) peak), β is the full width at half-maximum (FWHM) (β_c_ for (002) peak, β_a_ for (100) peak,), and N is the number of graphene layers [10]. Additionally, K, a constant dependent on crystal shape, was used as 0.89 for the (002) peak and 1.84 for the (100) peak [64,65].

In aerogels, the distance between plates is approximately 0.4 nm in the (002) plane and approximately 0.2 nm in the (100) plane. The number of layers in the (002) plane of aerogels is 4 on average. The crystal sizes (L_a_) of the aerogels vary between 4.8 and 6.4 nm according to Raman analyses, and between 6 and 7.9 nm according to XRD analyses.

Using XRD spectra, the graphitic structure amounts of the synthesized materials were also calculated. Equation 6 was used for these calculations [66].


(6) $$G\;(\%)=\frac{A_{1}}{A_{1}+A_{2}}\times 100$$

Here, G is the graphitic structure amount of the material and A1 is the peak area of the (002) plane. The location of A2 represents the graphitic residual peak area between the diffraction peaks of 2θ = 20° and 2θ = 26°. These fields are obtained by calculating the integral area of the peaks (two Gaussian peaks) representing amorphous and crystallized matter by fitting XRD curves [66,67]. The graphitic structure amount of aerogels is lower than GR and RGO, which are graphene-based precursor materials. This is due to the fact that the amorphism rate of GR and RGO, which have a 2D structure, is much lower than 3D graphene aerogels. The graphitic structure amount of GO is 61.7% due to the functional groups it contains. This result is also compatible with EDX analysis.

**Figure 8:**
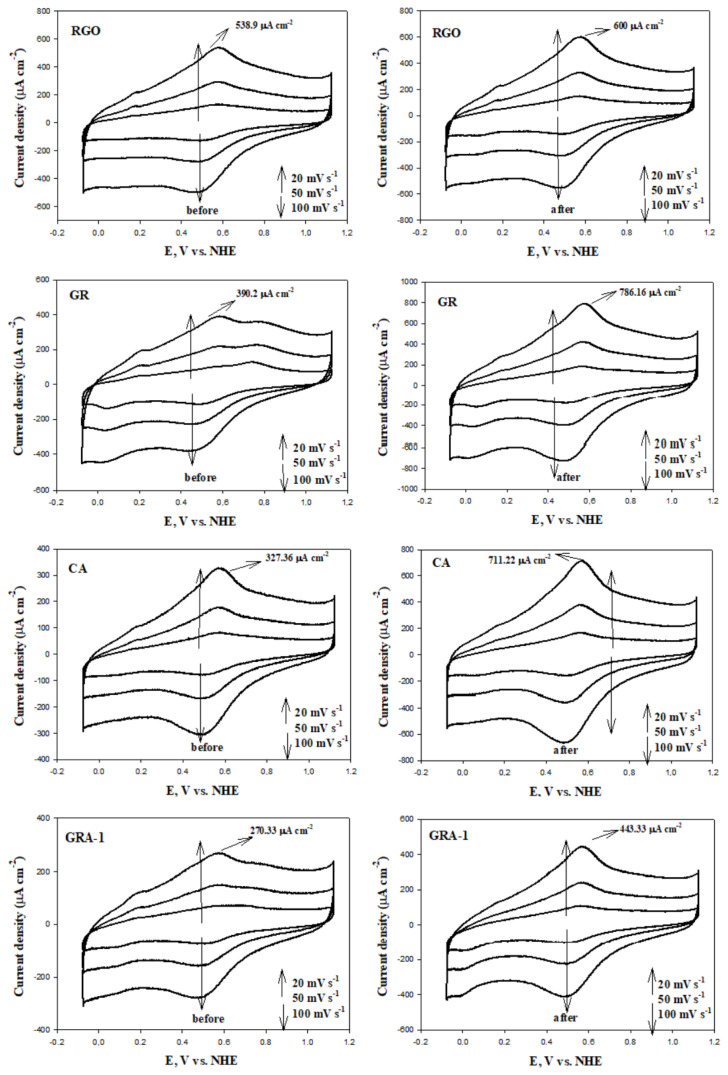

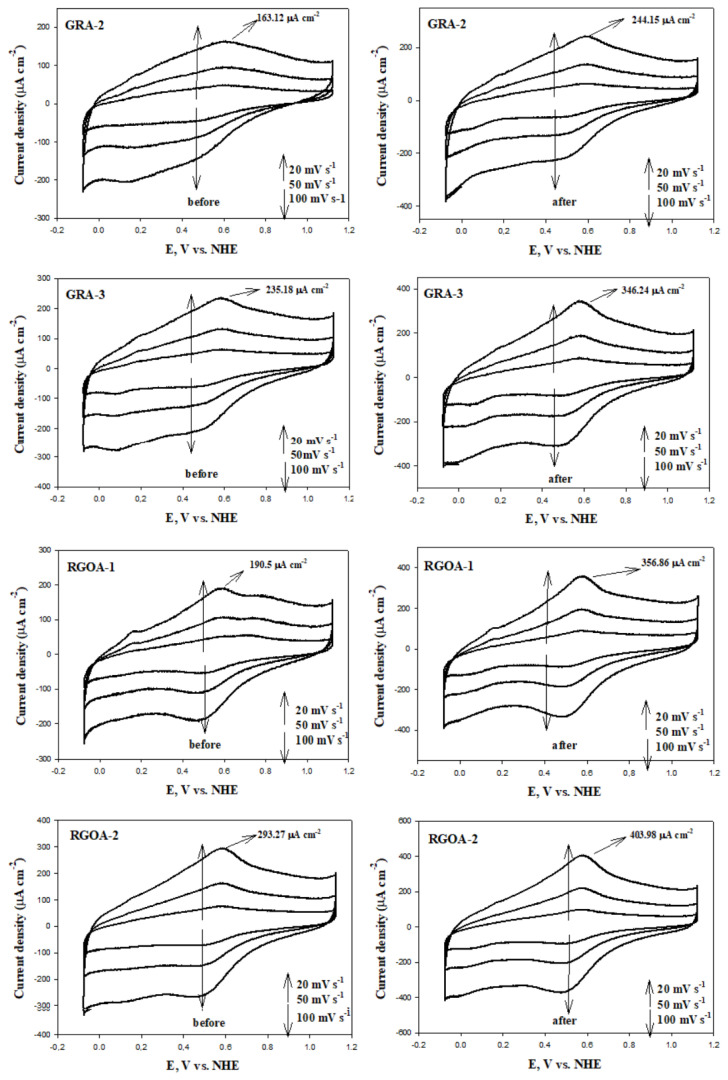

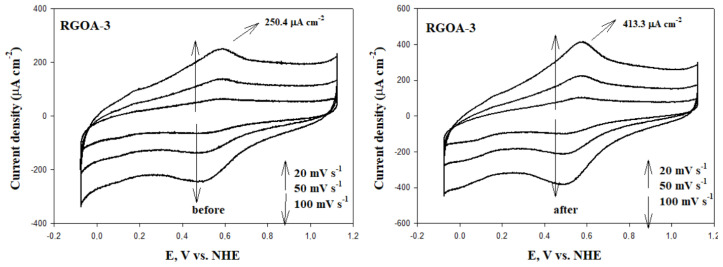
Cyclic voltammograms before and after carbon corrosion of RGO, GR, CA, and GAs (potential range: −0.28 to 0.92 V; scan rate: 20, 50, 100 mVs^−1^; electrolyte: N_2_ saturated 0.1 M HClO_4_ solution).

**Figure 9:**
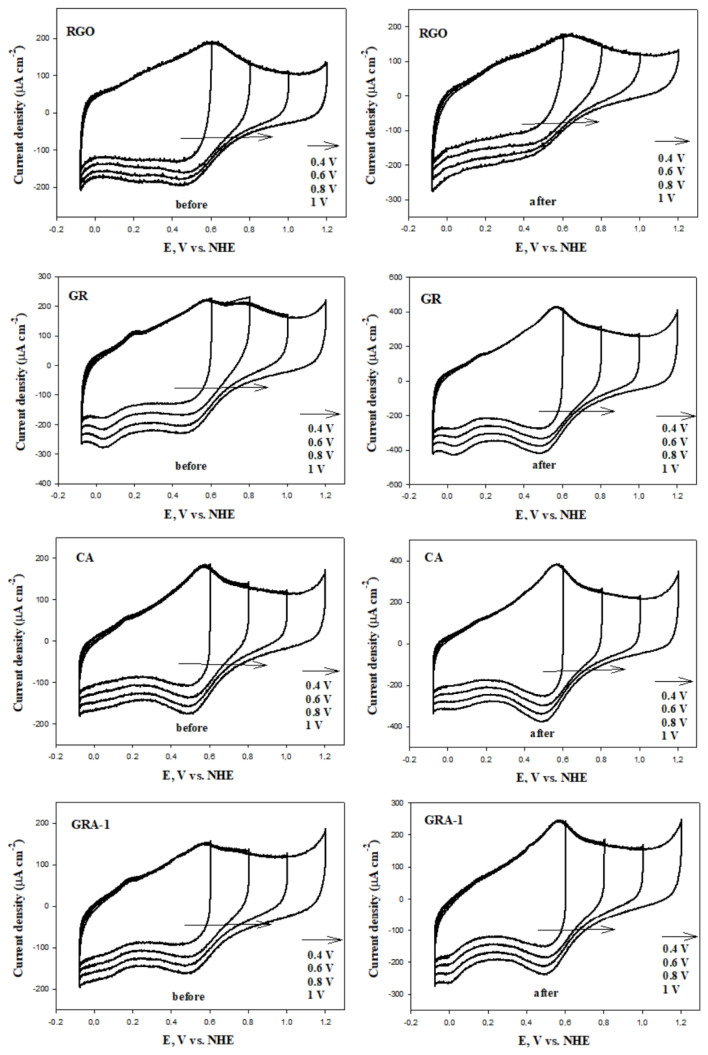

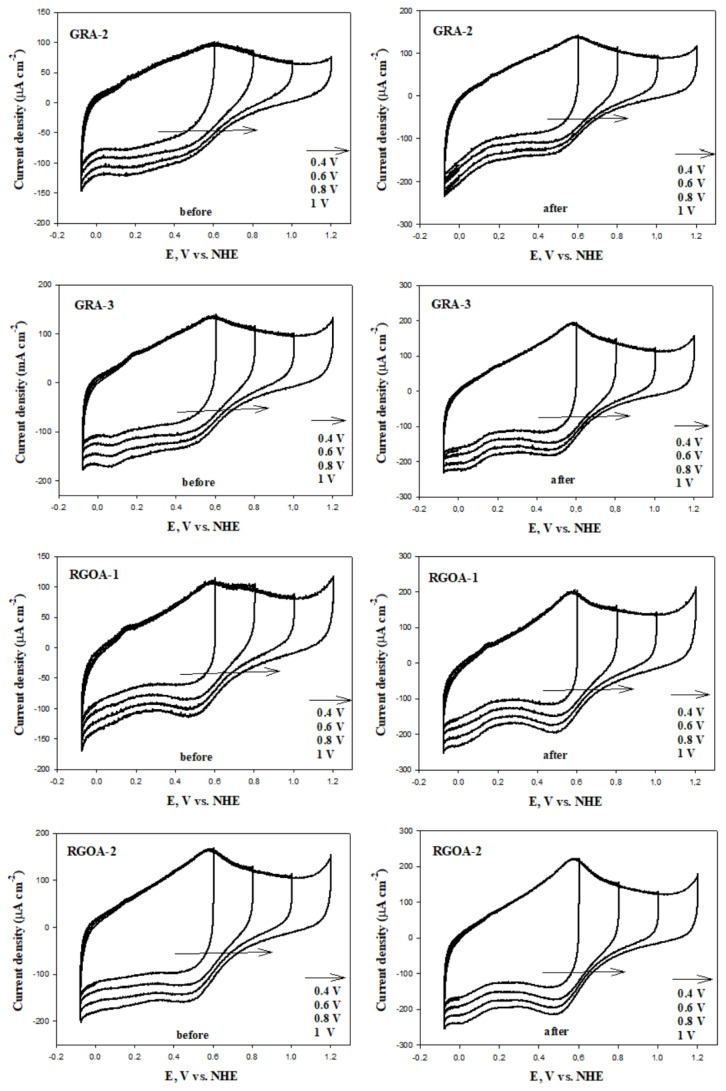

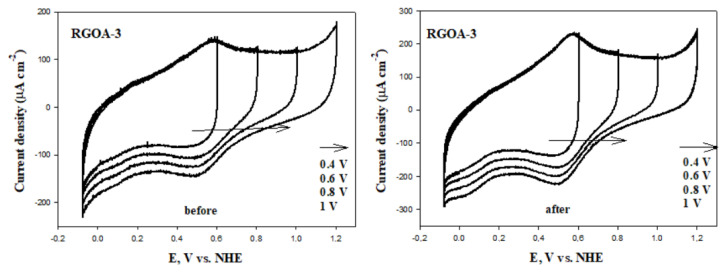
Cyclic voltammograms before and after carbon corrosion of RGO, GR, CA, and GAs (potential range: −0.28 to 0.4 V, −0.28 to 0.6 V, −0.28 to 0.8 V, −0.28 to 1 V; scan rate: 50 mVs^−1^; electrolyte: N_2_ saturated 0.1 M HClO_4_ solution).

The CV technique was used to study the electrochemical corrosion behavior of the surfaces of GR, RGO, CA, and GAs. The synthesized materials were subjected to corrosion for 24 h and CV measurements were taken before and after corrosion. CV voltammograms of electrochemical behavior at different scanning rates and different potential ranges are given in Figures 8 and 9, respectively. Although the mechanisms of corrosion on the carbon surface are not very clear and understandable, two reaction mechanisms are generally mentioned in some studies. The first of these is carbon→CO_2_, and the second is carbon→surface oxides [68]. The morphology, structure, and surface properties of carbonaceous materials strongly influence their behavior against electrochemical corrosion [69]. In their study, Cherstiouk et al. showed that current densities strongly depend on the microstructure of carbon materials and also increase with the ratio of grain boundaries between semigraphitic crystallites [69].

In the CV voltammograms of the different scanning rates given in Figure 8, it is seen that the upper current (anodic and cathodic current) values increase in the range of 400–800 mV as the scanning speed increases. This is due to the fact that the changes in the anodic and cathodic peaks due to the increasing internal resistance tend towards more positive and negative directions, respectively, as the scan rate increases at different scan rates [8]. The current values of the synthesized materials increased in the potential range of 400–800 mV after corrosion. This is due to the effects of quinone-hydroquinone (Q - HQ) redox pairs that occur to a large extent on the carbon surface during the oxidation process. When the increase rates of the upper flow values before and after corrosion are compared, it is seen that GAs increase less than GR and CA. This suggests that fewer surface oxide groups are formed after corrosion on the surface of GAs. Moreover, this is explained by the superior corrosion resistance of GAs compared to GR and CA without graphene.

In general, the electrochemical states occurring at the interface between the electrode and the electrolyte occur as two main processes. These are faradaic processes caused by redox reactions that occur with charge transfer between interfaces, and capacitive processes (adsorption/desorption stages) occurring in the electrical double layer (EDL) on the surface. Redox reactions and capacitive processes on the electrode surface occur only in certain potential ranges (cathodic and anodic potential barriers) and thermodynamical conditions are met, depending on the type of electrode and electrolyte used. Within these identified potential barriers, there is only a potential range where capacitive processes take place. This range is referred to as the electrochemical window or the EDL region of the system [70]. By using these intervals, researchers can investigate the redox reactions occurring on the electrode surface and conduct studies aimed at preventing corrosion effectively. The CV curves obtained at different potential ranges of the RGO, GR, CA, and GAs are given in Figure 9. As the potential upper limit increased, the CV curves took a rectangular shape depending on the electrical conductivity. This is due to the ideal double-layer capacitive behavior. In addition, peaks of anodic and cathodic currents have been observed due to pseudocapacitive effects arising from redox reactions occurring on the electrode surface within the range of 400–800 mV.

In carbon-based materials, the potential of the electrode constantly changes during charging/discharging processes. Electrochemically active surface groups can react at a certain potential or within potential ranges. Due to the complexity of organic compounds, there are also differences in the types of electrochemical reactions that occur in different potential ranges. That is, the potentials of redox reactions are determined by the type of surface groups. As long as these faradaic reactions can meet the criteria for “pseudocapacitance”, they have a positive effect on the “apparent capacitance”. Therefore, the apparent capacitance of an electrode is also different in different potential ranges [71].

**Figure 10:**
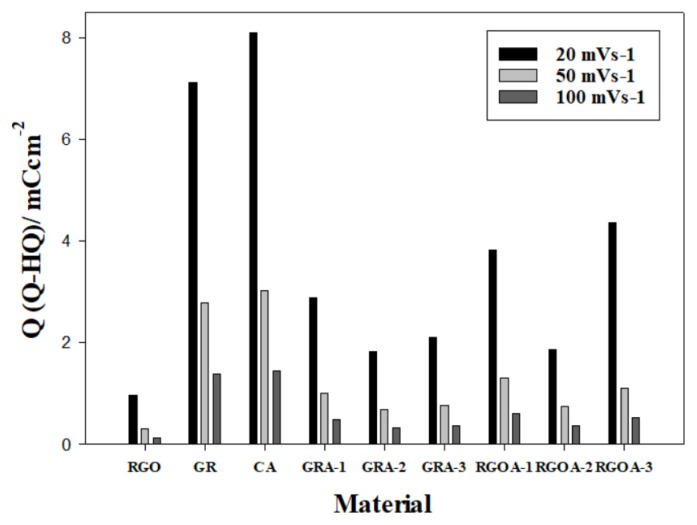
The amount of pseudocapacitive charge formed after corrosion on the surface of the synthesized materials.

Double-layer charging of carbon-based materials is used to monitor surface area changes, while pseudocapacitive charging is used to study the degree of surface oxidation (proportional to the faradaic charge passed in relation to the oxidation/reduction of redox-active surface functional groups) [72]. In other words, the charge change that occurs on the surface of the synthesized materials after corrosion gives the pseudocapacitive charge value resulting from Q - HQ redox reactions. The pseudocapacitive charge value also represents the corrosion rate [73]. For this reason, specific capacitance (C_P_) values before and after corrosion were calculated using CV voltammograms in the −0.28 to 0.92 V potential range and different scan rates (20, 50, 100 mVs^−1^) given in Figure 8. The results are summarized in Table 5. The electrochemical corrosion process caused an increase in C_P_ values. This difference is the pseudocapacitive charge formed on the surface. Pseudocapacitive charge values occurring after corrosion on the surface of the synthesized materials were calculated. The results are summarized in Table 5 and presented as a bar graph in Figure 10.

Specific capacitance values were calculated using equation 7 [1].


(7) $$C_{S}=\int \frac{\left(IdV\right)}{\left(vm\Delta V\right)}$$

Here, C_S_ is the specific capacitance, I is the current value, V is the potential window, ʋ is the scan rate, and m is the active mass used in the working electrode. The amount of charge occurring on the surface was calculated using equations 8 and 9 [74].


(8) $$C_{S}=\frac{dQ}{dV}=\frac{I}{\left(dV/dt\right)}$$

(9) $$Q(mC{cm}^{-2})=\int Idt=\int I/(dV/dt)dV$$

Here, Q is the charge, mC is the electric charge unit (millicoulomb), I is the current density (μAcm^−2^), and dV/dt is the scanning speed (mVs^−1^).

The highest C_P_ value belongs to RGO (279.74 Fg^−1^) and GR (249.11 Fg^−1^), which are graphene-based precursor materials. Among graphene aerogels, the highest capacitance value at 20 mVs^−1^ belongs to RGOA-3 (216.68 Fg^−1^). As the RGO ratio increased in RGOAs, the C_P_ value increased. However, the C_P_ value of GRAs decreased compared to CA and GR. However, the pseudocapacitance charge value of GRAs after corrosion decreased significantly compared to CA and GR. It is assumed that the decrease in the capacitive charge of carbon materials is related to corrosion resistance [75]. Therefore, the material with the highest postcorrosion resistance among aerogels is GRA-2, with a pseudocapacitive charge amount of 1.830 mCcm^−2^ at 20 mVs^−1^. The capacitive charge value is explained by the crystallinity, wettability, and porous size differences of the materials [75]. As the scan rate increased, the pseudocapacitive charge values decreased. This is due to the fact that at low scan rates, electrolyte ions have a higher chance of reaching the smallest and deepest pores [76]. Pseudocapacitive charge formation after corrosion of RGO is much lower than that of other materials. This is due to RGO’s large specific surface area, excellent mechanical properties and two-dimensional geometry [77]. Additionally, they are known to be less prone to electrochemical oxidation because they have a layered structure, fewer active/edge regions that would normally oxidize, and a more crystalline structure than aerogels [76].

**Figure 11:**
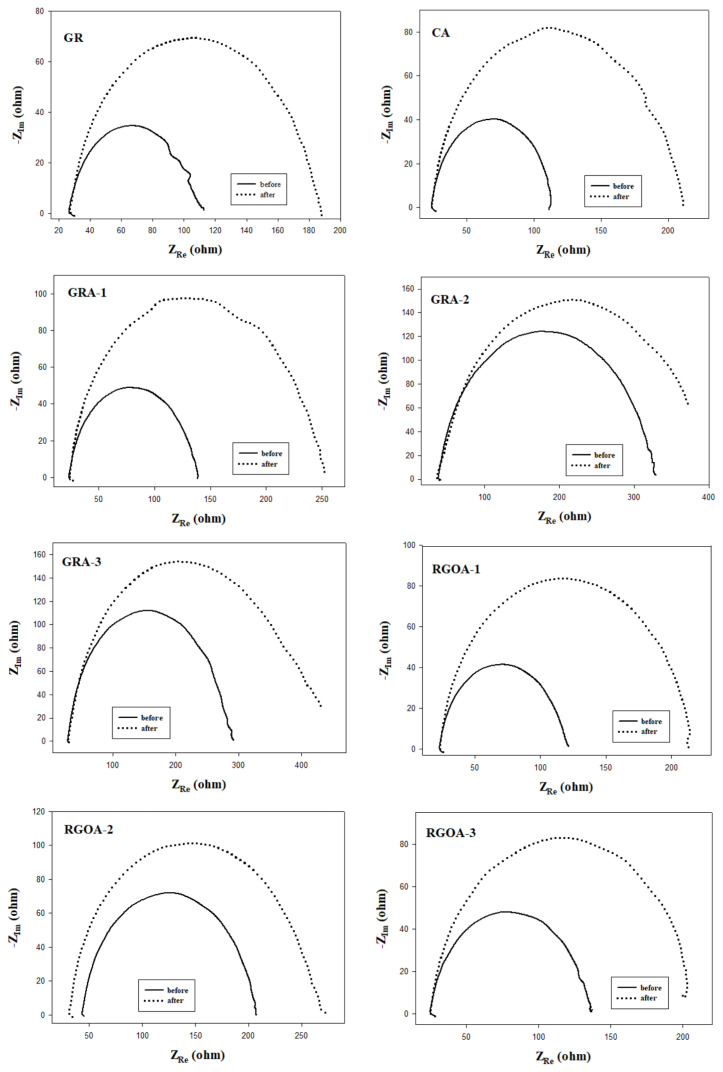

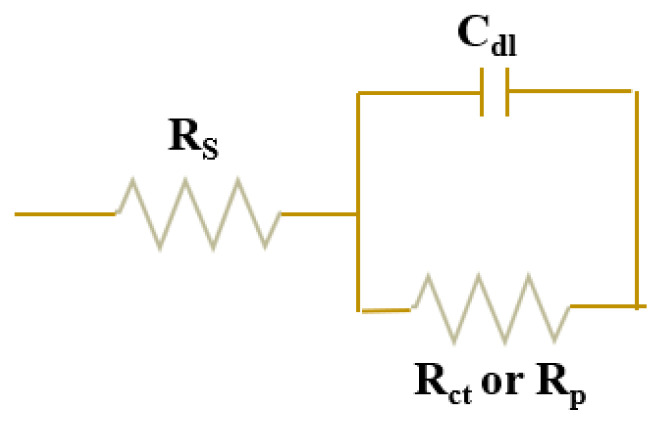
Nyquist plots before and after carbon corrosion (frequency range: 10^5^ to 1 Hz; potential: 0.9 V; N_2_ saturated 0.1 M HClO_4_ solution) and the electrical equivalent circuit model.

EIS analysis is an electrochemical technique used to analyze the internal resistance of the solution at the electrode/electrolyte interface, the charge transfer resistance, the electrical double-layer capacitance of the electrode, and the states occurring in the diffusion mass transfer process. Nyquist graphs were drawn using the data obtained from EIS analyses, and the equivalent circuit model that fits these graphs is given in Figure 11. Nyquist plots are compatible with the Randles equivalent circuit model. The Randles cell is a simple and convenient combination of a capacitor (double-layer capacitor (C_dl_)) and two resistors (solution (electrolyte) resistor (R_s_), charge transfer (R_ct_) or polarization resistor (R_p_)) [10]. The semicircle formed in the high- and mid-frequency region of the Nyquist plots represents the resistance due to the R_ct_. After corrosion, the charge transfer-induced resistance of all materials increased. This is due to the fact that the surfaces of the materials become more difficult to transfer as a result of oxidation after corrosion.

The numerical values of the data of Nyquist charts are summarized in Table 6. In Nyquist plots, the point where the high-frequency region intersects with the real impedance axis represents the R_s_, the point where the low-frequency region intersects with the real impedance axis represents the R_ct_, and the diameter of the semicircular impedance curves represents the R_p_ [10,78]. Since the electrolyte used in the analysis medium is the same, the R_s_ values of all materials are close to each other. The materials with the highest R_ct_ before corrosion are RGO, GRA-2, and GRA-3. After corrosion, an increase was observed in R_ct_ values, and accordingly, R_p_ values. The materials with the highest rate of increase in R_p_ values after corrosion are GR, CA, GRA-1, and RGOA-1. The GAs least affected by corrosion are those containing 0.5% and 1% graphene-based materials.

## 5. Conclusions

Carbonaceous aerogels have attracted the attention of many researchers due to their superior physical and chemical properties. In this study, the synthesis and structural characterization of GAs, the most important class of carbonaceous aerogels, is reported. While GO was used as a graphene-based precursor material in GA synthesis in the literature, commercial GR and chemical RGO were used at different rates in this report, unlike the literature. The sol-gel method was used in the synthesis of GAs and was carried out in three stages: gelation, SCCO_2_ drying, and pyrolysis stages. The differences in the surface properties of graphene-based precursor materials (GR, RGO) significantly changed the cross-linking mechanism of the aerogel in the gelation stage. As a result, a wide variety of GAs with very different properties has been synthesized, as can be clearly seen with SEM images. As the GR ratio increased in GRAs, the BET surface area increased, while the BET surface area decreased as the RGO ratio increased in RGOAs. The highest surface area belongs to RGOA-1 with 511.75 m^2^g^−1^. CV and EIS analyses were performed to examine the behavior of GAs against electrochemical corrosion. After the corrosion of the synthesized materials, the charge transfer resistance at the electrode-electrolyte interface increased. Capacitance values of the materials synthesized before corrosion were calculated. The highest C_P_ value belongs to RGO (279.74 Fg^−1^) and GR (249.11 Fg^−1^), which are graphene-based precursor materials. Among graphene aerogels, the highest capacitance value at 20 mVs^−1^ belongs to RGOA-3 (216.68 Fg^−1^). Among the aerogels, the material with the highest resistance after the electrochemical corrosion process is GRA-2, with a pseudocapacitive charge amount of 1.830 mCcm^−2^ at 20 mVs^−1^. The corrosion resistance of the electrode material used in systems where energy is stored and converted is one of the most important parameters. In this study, the corrosion resistance of graphene-based materials and various graphene aerogels as electrode materials was examined. In our future studies, it is aimed to use these synthesized materials as support materials as the electrocatalyst in PEMFCs.

## Figures and Tables

**Table 1:** BET results of graphene-based materials and Gas.

Material	BET surface area (m^2^g^−1^)	Average pore diameter (nm)	t-Plot Micro pore area (m^2^/g)	t-Plot Micro pore volume (cm^3^g^−^^1^)	Average particle size (nm)	BJH pore volume (cm^3^g^−^^1^)	D-H pore volume (cm^3^g^−^^1^)
**Graphite** [10]	25.25	5.51	--	--	237.64	0.053	0.063
**GO** [10]	10.51	4.85	--	--	570.93	0.016	0.020
**RGO** [10]	166.92	4.27	--	--	35.95	0.205	0.259
**GR**	710.34	5.63	113.57	0.054	8.45	1.189	1.176
**CA**	202.86	2.91	115.72	0.050	29.58	0.108	0.106
**GRA-1**	172.16	4.38	94.83	0.047	34.85	0.193	0.191
**GRA-2**	185.43	3.91	118.09	0.059	32.36	0.158	0.156
**GRA-3**	471.88	4.46	250.09	0.129	12.72	0.44	0.435
**RGOA-1**	511.75	3.57	174.72	0.086	11.72	0.341	0.337
**RGOA-2**	378.28	3.62	160.50	0.080	15.86	0,268	0.265
**RGOA-3**	224.75	2.65	122.13	0.061	26.69	0.073	0.072

**Table 2:** The C/O ratios of graphene-based materials and Gas.

Material	C/O	
	Weight	Atomic
**RGO**	1.93	2.57
**GR**	7.40	9.87
**CA**	16.45	21.94
**GRA-1**	8.33	11.09
**GRA-2**	18.92	25.25
**GRA-3**	10.99	14.63
**RGOA-1**	20.46	27.25
**RGOA-2**	8.35	11.12
**RGOA-3**	7.73	10.29

**Table 3:** Peak and band analysis of GR, CA, GRAs, and RGOAs to Raman spectra.

Material	Raman shift (cm^−^^1^)		D peak intensity (a.u.)	G peak intensity (a.u)	I_D_/I_G_	L_a_ (Å)
	D band	G band				
**Graphite** [10]	1333.87	1562.1	847.5	1347.75	0.63	69.8
**GO** [10]	1347.35	1606.8	1932	1674	1.15	38.3
**RGO** [10]	1351.41	1598.94	889.00	750.50	1.18	37.3
**CA**	1326.76	1580.78	613.72	835.73	0.73	60.3
**GR**	1326.76	1559.44	477.34	880.78	0.54	81.5
**GRA-1**	1326.76	1602.11	1252.30	1358.20	0.92	47.8
**GRA-2**	1305.43	1580.78	2116.80	2574.10	0.82	53.7
**GRA-3**	1326.76	1580.78	1543.10	1748.50	0.88	50.0
**RGOA-1**	1369.44	1580.78	536.50	627.94	0.85	51.8
**RGOA-2**	1326.76	1580.78	670.65	970.35	0.69	63.8
**RGOA-3**	1326.76	1580.78	762.28	903.18	0.84	52.4

**Table 4:** Data of XRD spectra of GR, CA, GRAs, and RGOAs.

Material	(002)				(100)				G (%)
	2θ	d (Å)	L_c_ (Å)	N	2θ	d (Å)	L_a_ (Å)	N	
**Graphite** [10]	26.70°	3.3	58.5	17.5	--	--	--	--	100
**GO** [10]	11.40°	7.8	22.6	2.9	--	--	--	--	61.7
**RGO** [10]	22.90°	3.9	18.1	4.6	--	--	--	--	78.6
**GR**	26.21°	3.4	25.4	7.5	43.38°	2.09	127.7	61.1	85.6
**CA**	22.00°	4.0	15.8	4.0	43.79°	2.07	63.7	30.8	73.0
**GRA-1**	22.41°	3.9	15.4	3.9	43.58°	2.37	63.5	26.8	73.1
**GRA-2**	22.63°	3.9	15.8	4.1	43.79°	2.07	66.1	31.9	75.7
**GRA-3**	22.21°	4.0	16.1	4.0	43.58°	2.08	59.9	28.8	72.2
**RGOA-1**	21.58°	4.1	17.1	4.2	43.79°	2.07	78.8	38.1	71.8
**RGOA-2**	21.35°	4.2	15.7	3.7	43.79°	2.07	78.7	38.0	70.8
**RGOA-3**	22.41°	3.9	16.1	4.1	43.58°	2.08	66.1	31.8	73.4

**Table 5:** Specific capacitance and pseudocapacitive charge values of the synthesized materials.

Material	Before corrosion C_S_ (Fg^−^^1^)			After corrosion C_S_ (Fg^−^^1^)			Pseudocapacitive charge Q (mCcm^−^^2^)		
	20 mVs^−^^1^	50 mVs^−^^1^	100 mVs^−^^1^	20 mVs^−^^1^	50 mVs^−^^1^	100 mVs^−^^1^	20 mVs^−^^1^	50 mVs^−^^1^	100 mVs^−^^1^
**RGO**	279.74	247.14	225.50	299.00	262.17	238.80	0.963	0.300	0.133
**GR**	249.11	207.79	180.07	391.52	346.69	318.95	7.120	2.778	1.390
**CA**	166.62	141.78	128.67	328.75	293.07	273.00	8.110	3.026	1.443
**GRA-1**	165.13	140.65	125.53	222.66	191.28	174.33	2.877	1.013	0.488
**GRA-2**	107.06	87.27	75.32	144.20	121.94	108.79	1.830	0.693	0.335
**GRA-3**	141.22	120.45	107.59	183.21	158.7	144.09	2.100	0.765	0.365
**RGOA-1**	114.84	95.76	84.28	191.28	161.39	145.00	3.822	1.313	0.607
**RGOA-2**	164.38	139.27	125.53	201.74	176.93	162.30	1.868	0.753	0.368
**RGOA-3**	216.68	128.52	114.17	303.95	183.63	165.87	4.364	1.102	0.517

**Table 6:** The electrolyte resistance (R_s_), the charge transfer resistance (R_ct_), and the polarization resistance (R_p_) of the materials before and after carbon corrosion.

Materials	Before corrosion			After corrosion			% R_P_ increase rate
	R_s_ (Ω)	R_ct_ (Ω)	R_p_ (Ω)	R_s_ (Ω)	R_ct_ (Ω)	R_p_ (Ω)	
**RGO** [10]	27.1	259.37	232.27	26.94	436.7	409.76	76
**GR**	29.79	112.77	82.98	29.82	188.17	158.35	91
**CA**	26.81	111.49	84.68	26.82	210.44	183.62	117
**GRA-1**	25.03	138.62	113.59	27.08	250.09	223.01	96
**GRA-2**	41.57	329.66	288.09	39.66	374.41	334.75	16
**GRA-3**	30.55	292.31	261.76	30.44	431.32	400.88	53
**RGOA-1**	24.78	121.79	97.01	25.76	212.59	186.83	93
**RGOA-2**	45.35	207.41	162.06	33.94	272.29	238.35	47
**RGOA-3**	28.72	137.18	108.46	28.19	199.71	171.52	58
